# Aptasensors Are Conjectured as Promising ALT and AST Diagnostic Tools for the Early Diagnosis of Acute Liver Injury

**DOI:** 10.3390/life13061273

**Published:** 2023-05-29

**Authors:** Raja Chinnappan, Tanveer Ahmad Mir, Sulaiman Alsalameh, Tariq Makhzoum, Salma Adeeb, Khaled Al-Kattan, Ahmed Yaqinuddin

**Affiliations:** 1 College of Medicine, Alfaisal University, Riyadh 11533, Saudi Arabia; salsalameh@alfaisal.edu (S.A.); tmakhzoum@alfaisal.edu (T.M.); snadeeb@alfaisal.edu (S.A.); kkattan@alfaisal.edu (K.A.-K.); 2 Tissue/Organ Bioengineering & BioMEMS Lab, Organ Transplant Centre of Excellence, Transplant Research & Innovation Department, King Faisal Specialist Hospital and Research Centre, Riyadh 11211, Saudi Arabia; tmir@kfshrc.edu.sa

**Keywords:** alanine aminotransferase (ALT), aspartate aminotransferase (AST), aptamers, aptasensors, SELEX, lateral flow assay, liver injury

## Abstract

Abnormal levels of alanine aminotransferase (ALT) and aspartate aminotransferase (AST) in human serum are the most sensitive indicator of hepatocellular damage. Because liver-related health problems are directly linked to elevated levels of ALT and AST, it is important to develop accurate and rapid methods to detect these enzymes for the early diagnosis of liver disease and prevention of long-term liver damage. Several analytical methods have been developed for the detection of ALT and AST. However, these methods are based on complex mechanisms and require bulky instruments and laboratories, making them unsuitable for point-of-care application or in-house testing. Lateral flow assay (LFA)-based biosensors, on the other hand, provide rapid, accurate, and reliable results, are easy to operate, and are affordable for low-income populations. However, due to the storage, stability, batch-to-batch variations, and error margins, antibody-based LFAs are considered unaffordable for field applications. In this hypothesis, we propose the selection of aptamers with high affinity and specificity for the liver biomarkers ALT and AST to build an efficient LFA device for point-of-care applications. Though the aptamer-based LFA would be semiquantitative for ALT and AST, it would be an inexpensive option for the early detection and diagnosis of liver disease. Aptamer-based LFA is anticipated to minimize the economic burden. It can also be used for routine liver function tests regardless of the economic situation in each country. By developing a low-cost testing platform, millions of patients suffering from liver disease can be saved.

## 1. Introduction

Chronic liver disease is a global health-threatening issue that is difficult to diagnose and treat on time due to a lack of early and accurate detection strategies. Untreated chronic liver diseases such as hepatitis B and C can lead to cirrhosis and liver cancer with substantial short-term mortality [1,2]. Alanine aminotransferase (ALT) and aspartate aminotransferase (AST) are important biomarkers to evaluate liver functions [3,4]. Conventional detection methods such as spectrophotometric, fluorometric enzyme-linked immunosorbent assay (ELISA), and kinetic enzymatic assay for the diagnosis of ALT and AST are not very sensitive and require laboratories with expensive bulk instruments [5]. Therefore, these methods are not suitable for point-of-care (POC) applications or home use in self-health monitoring. In recent years, biosensors have evolved as an emerging tool capable of detecting biomarkers of specific diseases. These miniaturized user-friendly biosensor devices are more specific, sensitive, and rapid, have low production costs, and can be used in POC settings [6,7,8]. Biosensors integrate an analyte recognition element, a transducer, and a signal amplifier. Monoclonal antibodies (mAbs) play a major role in modern medicine, and they are often used as the recognition element in most immunosensor devices [9]. However, they have certain drawbacks such as stability, sensitivity, and production cost.

Nucleic-acid-based aptamers are emerging recognition receptor alternatives to antibodies for the development of various biomedical diagnostics and therapeutic methods. Aptamers have many advantages over antibodies, including greater stability, easy production, and chemical modification [10]. Aptamers are single-stranded DNA or RNA oligonucleotides, and their unique conformation allows them to bind to target analytes with high affinity and specificity [11]. Aptamers are selected by an in vitro screening method known as systematic evolution of ligands by exponential enrichment (SELEX). Several aptamer strands have been selected against a plethora of molecules, including toxins [12], allergens [13], drugs [14], hormones [15], and whole cells [16], exhibiting high sensitivity and selectivity. Aptamer-based biosensors (aptasensors) exploit aptamers as bioreceptors that specifically bind the target, followed by a translation of the output information into a measurable signal via a transducer. Therefore, they are widely used in biosensing applications to identify biological components or biomarkers for diagnosing different diseases. An ideal POC device must be affordable, sensitive, specific, user-friendly, rapid and robust, equipment-free, and deliverable (ASSURED). A number of aptasensors for POC applications have been developed to detect various diseases [17,18,19,20]. For example, hepatitis B is an infectious disease that causes liver dysfunction. Fluorescence-based aptasensors have been developed for the detection of hepatitis B antigens. In this study, a fluorescence-labeled HBeAg-specific aptamer was employed as a molecular recognition element, and the short complementary oligonucleotides were used as a competitor for the target [21]. However, aptamers for ALT and AST (more specific and accurate biomarkers of liver injury) have not been reported. We propose the development of a POC test using aptamers to detect ALT and AST levels in biological fluids as a superior liver function test.

**Hypothesis** **1** **(H1).** *Selection of highly sensitive, specific aptamers for ALT and AST by SELEX procedure.*

**Scheme 1:**
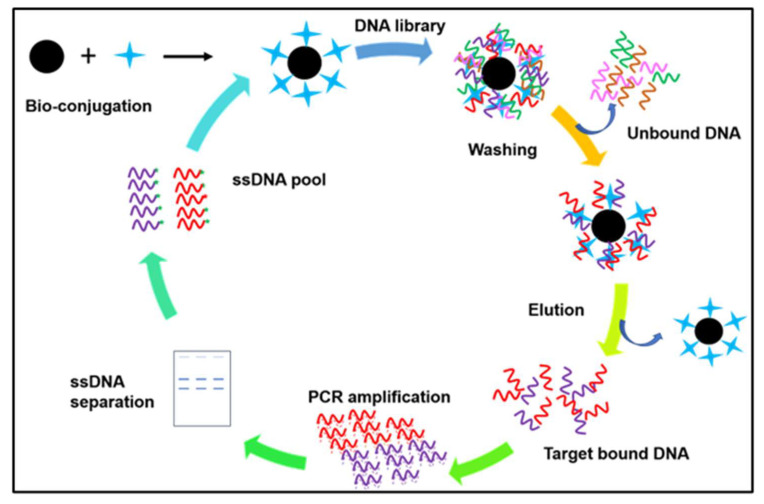
Working principle of aptamer selection by SELEX procedure.

An efficient POC diagnostic testing device for ALT and AST levels for monitoring acute liver injury from the blood or serum samples should be rapid and robust, without the need for additional equipment and trained professionals. As aptamers are an ideal candidate for ALT and AST recognition receptors, we propose the selection of highly specific, selective aptamers to design an aptamer-based POC testing device. To the best of our knowledge, there are no aptamers selected against ALT and AST enzymes. Integration of thermally stable anti-ALT and anti-AST aptamers in the POC testing device will reduce fluctuation in the readout and minimize false positives and negatives [22]. Generally, aptamers are selected against pure analytes, where any contamination in the sample leads to the selection of aptamers for the contaminants as well. In this proposed hypothesis, anti-ALT and anti-AST aptamers will be selected via SELEX. ALT and AST enzymes are immobilized on the solid surface or physically adsorbed on 96-well plates in physiological conditions. After washing, the free enzymes are removed, leaving immobilized/adsorbed enzymes for aptamer selection. A synthetic ssDNA library of 10^15^ sequences is incubated with the analyte under optimal conditions. The typical library sequences consist of 30–60 random nucleotides with two fixed-terminal primer binding sites on both the 5′ and 3′ ends. The forward primer is labeled with fluorescein at the 5′ end, while the reverse primer is labeled with poly-A along with hexa-ethylene glycol spacer at the 3′ end. The fluorescein forward primer acts as a probe for the quantification of the DNA recovery during each round of the selection step. The reverse primer has a hexa-ethylene glycol spacer and poly-A at the 5′ end. The hexa-ethylene glycol spacer does not allow the polymerase to extend the PCR product and stop the amplification reaction. The PCR amplification reaction with these two primers leads to the formation of a double-stranded DNA product where each DNA strand has different lengths. When the PCR products are employed to denaturing polyacrylamide gel electrophoresis (PAGE), the two strands with different lengths are separated. The ssDNA collected from the 4th or 5th cycle is incubated with blank sepharose beads to remove any non-specific and low-binding DNA sequences (counter selection). After the completion of about 12 SELEX cycles, the eluted ssDNA is amplified using unlabeled primers, and the PCR product is used for ligation and cloning. Schematic representation of all the SELEX processes are shown in the Scheme 1. The double-stranded PCR product is ligated into the pCR2.1-TOPO cloning vector. Then, the ligated product is transformed into *E. coli DH5α*-competent cells. The positive colonies are picked up, and colony PCR is performed for each colony using M13 forward and reverse primers to amplify the ssDNA inserts. The PCR products are then sequenced to identify the binding affinity of each aptamer with their corresponding targets (ALT or AST). The binding affinity of the selected aptamers against ALT and AST will be confirmed using fluorescence assay. Variable concentrations of labeled aptamers are incubated with ALT/AST-conjugated beads. After removing unbound aptamers, the fluorescence intensity of each eluted fraction is measured and correlated to the quantity of the recovered DNA. Binding affinity curves are obtained from the plot of the ssDNA aptamer concentration versus the corresponding fluorescence intensity for each aptamer sequence. The dissociation constant (K_d_) is determined via nonlinear regression analysis of the hyperbolic curve. The high-affinity aptamers are used to develop aptasensors.

Although aptamers have high selectivity and specificity, there are many challenges in sequentially selecting aptamers through the SELEX process. It is a common practice to use monoclonal and/or polyclonal antibodies for clinical applications. However, batch-to-batch variation in antibody production is a major pitfall that can be resolved by aptamer-based clinical analysis. In addition, aptamers can be selected against a wide spectrum of targets that are non-immunogenic or toxic. On the other hand, antibody production requires in vivo screening, and its targets elicit strong reactions from the immune system. Therefore, it is extremely difficult for antibody-based approaches to recognize and capture toxic or non-immunogenic molecules. Despite the many advantages, their clinical utility for therapeutic purposes is still limited due to their susceptibility to serum nucleases, swift elimination by renal filtration, and low binding affinity to their targets in vivo. Specificity and cross-reactivity are also major obstacles to the use of aptamers for biodetection in clinical samples, as aptamers selected for their targets may also bind to closely related or structurally similar molecules. For example, aptamers selected against DNA polymerase enzyme β also showed binding ability against DNA polymerase enzyme κ [23]. These problems can be solved by negative selection against the closely related targets during the SELEX protocol. Another major concern is the generation of aptamers against the purified proteins using conventional SELEX procedures. For example, aptamers selected against recombinant proteins expressed in cell culture methods or prokaryotic cells and affinity-purified proteins may not be usable for real sample analysis. In addition, if the conformation of the purified protein differs from that of the native protein, the binding pocket of the aptamer may not fit the native protein, and the aptamer may not recognize the target protein [24]. For example, RNA aptamers for the histidine-tagged EGFRvIII ectodomain were expressed and purified using E. coli and sorted in vitro. Despite the high affinity and specificity of the aptamer against the target, it did not bind to the same protein expressed from eukaryotic cells due to the post-translational modification (glycosylation) in the EGFRvIII protein. This is due to the differences in the structural conformation of the protein used for the aptamer selection [25]. Since biomarker proteins are expressed on the cell surfaces, aptamer libraries can bind directly to healthy or viable cell surfaces without protein purification steps, indicating that the whole-cell SELEX method can be useful for the efficient selection of target-specific aptamers for routine clinical sample analysis. In addition, no prior knowledge of specific outer membrane proteins is required, and dead cells can be removed by fluorescence-activated cell sorting techniques [26,27].

### 1.1. Evaluation and Outcome of Hypothesis: ALT and AST Biosensors/Aptasensors as Self-Health and Liver Function Monitors

Biosensors for detecting biomarkers of liver diseases are in constant demand. A number of biosensors have been developed for the detection of ALT and AST. Several detection techniques have been applied for developing biosensors. Aptasensors are a type of biosensors in which aptamers are exploited as target recognition elements. Aptamers exhibit an extraordinary role in clinical diagnostic assay development, as they can be chemically modified with functionalized molecules for immobilization or probing, without affecting the affinity, using various methods [28,29]. Moreover, aptamers are thermally stable and therefore can be engineered in such a way that they regenerate their bioreceptor function after many repeated cycles. Since the patient’s biomarker levels correlate with their health status, aptasensors have the potential to take on a unique role in biomarker detection and patient monitoring. A review from Bharadwaj and Sharma has demonstrated the utility of aptasensors in routine full-body check-ups [30]. Aptasensors were used to detect potential biomarkers to monitor patients’ sugar profile, cardiac risk profile, vitamin level, kidney profile, liver profile, lipid profile, urine analysis, inflammation markers profile, blood element profile, serum electrolytes profile, and hormone profile. Out of the many liver biomarkers, such as ALT, AST, alkaline phosphatase (ALP), gamma-glutamyltransferase (GGT), L-lactate dehydrogenase (LD), bilirubin, and human serum albumin (HAS) for liver functions, aptamers are reported only for HSA [31]. Since all liver biomarkers (except for HSA) are enzymes, their enzymatic activity can be exploited for their detection. Nevertheless, using aptamers’ unique advantages for developing ASSURED devices using ALT and AST as liver functional biomarkers has great potential for developing a low-cost POC assay. An anti-transforming growth factor-beta-1 (TGF-β1) aptamer-based sensor has been designed for the detection of liver fibrosis through TGF-β1 monitoring. The cultured biosensor involved the integration of gold electrodes and reconfigurable microfluidics. A thiolated aptamer with a methylene blue redox reporter was self-assembled on a gold electrode surface in a PDMS microfluidic chip. In the absence of a target, a greater current was generated due to methylene blue’s proximity to the electrode surface. Upon target binding, the aptamer underwent a conformational change, drifted the methylene away from the electrode surface, and reduced the current. The aptasensor was validated with stellate cells, and the limit of detection was 1 ng/mL [32]. However, TGF-β1 is one of the biomarkers of liver function but is not a more specific marker for liver injury. Therefore, aptamers were selected against ALT and AST to be used for the creation of ASSURED devices for point-of-care applications, as demonstrated for thrombin detection [33,34]. In an electrochemical study, pyruvate oxidase was immobilized onto an electrode, with poly(4-aminophenol) and 4-aminoantipyrine used as redox indicators. In the presence of ALT, alanine, L-alanine, and α-ketoglutarate produced pyruvate, which was further oxidized by pyruvate oxidase (PyO). The H_2_O_2_ produced was reacted with 4-aminoantipyrine, which generated electrochemical signals. This method utilized the enzymatic activity of ALT and could detect ALT as low as 2.68 × 10^−5^ U/L [35].

Xuan et al. developed electrochemical immunosensors for ALT sensing. This sensor consists of three layers, comprising an anti-ALT antibody-immobilized outer membrane layer and an inner membrane layer adsorbed with PyO. In the third layer, there was a self-assembled monolayer mediator-coated gold working electrode and a Ag/AgCl reference electrode. These immunosensors could detect ALT with an LOD of 10 pg/mL without any pre-washing within 5 min [36]. Real-time electrochemical-luminescent biosensors for ALT detection were demonstrated by Chu et al. They use the enzymatic oxidation of pyruvate by PyO, which releases H_2_O_2_ and subsequently produces electrochemical luminescence in the presence of luminol. Their rapid response and real-time detection of ALT in the dynamic range of 0.00475 to 350 U/L make them potential devices for monitoring liver function [37]. In another biosensor, an array of electrochemical sensors were developed using microelectromechanical systems (MEMS) for the rapid detection of ALT/AST using porous silicon layers with a high surface area for its working electrode. They used a platinum electrode and polydimethylsiloxane (PDMS) microchannel for the biosensor array to simultaneously detect ALT and AST. The sensitivities for ALT and AST were 0.145 U/L and 0.463 U/L, respectively [38]. In another electrochemical study, an iridium-carbon nanoparticles-based biosensor was fabricated for the detection of ALT. The biosensor mechanism quantified the H_2_O_2_ concentration generated from ALT enzymatic reactions. The sensor functioned well in PBS buffer, calf serum, and human serum samples; it was tested in the concentration range of 0–544 ng/mL, and the LOD was 2.18 U/L [39]. Lai et al. developed a point-of-care testing (POCT) device for ALT based on a dry chemistry-based electrochemiluminescence (DC-ECL) chip. The chip device includes the integration of two parts. The first part includes an outer shell (top cap and pedestal) and detection layer (baseplate, electrode pad, and carrier pad). The second part includes an automatic ECL analyzer associated with other counterparts. The sensor signals were measured in the range of 5–50 U/L with an LOD of 1.702 U/L [40] Tian et al. utilized the peroxidase-like activity of a B-doped core–shell Fe@BC nanozyme for the detection of ALT from the enzymatic activity. The H_2_O_2_ generated from the ALT enzymatic reaction oxidized 3,3′,5,5′-tetramethylbenzidine (TMB) and produced blue-colored oxTMB. The absorbance at 450 nm was used for the quantification of ALT. The LOD of the sensor was found to be 4 U/L from the signal measured in the range of 10–1000 U/L. The results from this method were compared with the standard methods used in hospitals [41]. In another electrochemical sensing device, both ALT and AST were detected, in which the electrode was modified with pyruvate oxidase (POX) and oxaloacetate decarboxylase. In the presence of suitable substrates, ALT and AST undergo enzymatic reactions and, as a result, changes in the electrical signals. The ALT and AST activities were proportional to the strength of signal change, which could be quantified. The device was tested in the range of 7.5–720 U/L with an LOD of 13.8 U/L and 13.9 U/L in PBS for ALT and AST, respectively [42]. Hsueh et al. fabricated an electrochemical biosensor consisting of catalytic iridium nanoparticles dispersed on carbon paste for the detection of AST. The amount of enzymatically produced H_2_O_2_ was used for the quantification AST, and the LOD of the device was 25.3 U/L [43]. In another study, Her et al. designed and fabricated a label-free ALT detection method using a graphene field-effect transistor. This device was made using a large area of a graphene thin film attached with L-alanine and α-ketoglutarate-immobilized in an alginate hydrogel, which favored detecting ALT at low voltage [44]. Various biosensing methods developed for the detction of ALT ans ASt are summarized in the Table 1. All the above-mentioned biosensors are based on detecting the enzymatic activity of ALT and AST as opposed to the protein itself. Antibodies or enzymes were used for the biosensor component in these devices.

High-affinity, short, and unsaturated peptides were selected from the M13 phage display for the development of ALT biosensors. Biopanning of the M13 phage display library, with immobilized ALT, leads to the selection of high-affinity peptides towards ALT. In one study, Wu et al. developed biosensors for ALT detection using electrochemical impedance spectroscopy (EIS) and quartz crystal microbalance (QCM), with peptides exploited as biorecognition receptors. The LOD of ALT was 92 ng/mL and 62 ng/mL for EIS and QCM, respectively [45]. This method was very straightforward and did not require ALT substrate, coenzymes, and other reagents. While aptasensors share similar working principles, they are superior to peptides in robustness. Antibodies and enzymes are not thermally stable, losing their activity over time, and the cost of production is high, which leads to variations between batches. Aptamers are chemical antibodies that lack these limitations, making them candidates for use in portable POC diagnostic devices.

### 1.2. Consequences of Hypothesis and Discussion: Aptasensors as Rapid Point-of-Care Detection of ALT/AST and Self-Health Monitor

**Figure 1:**
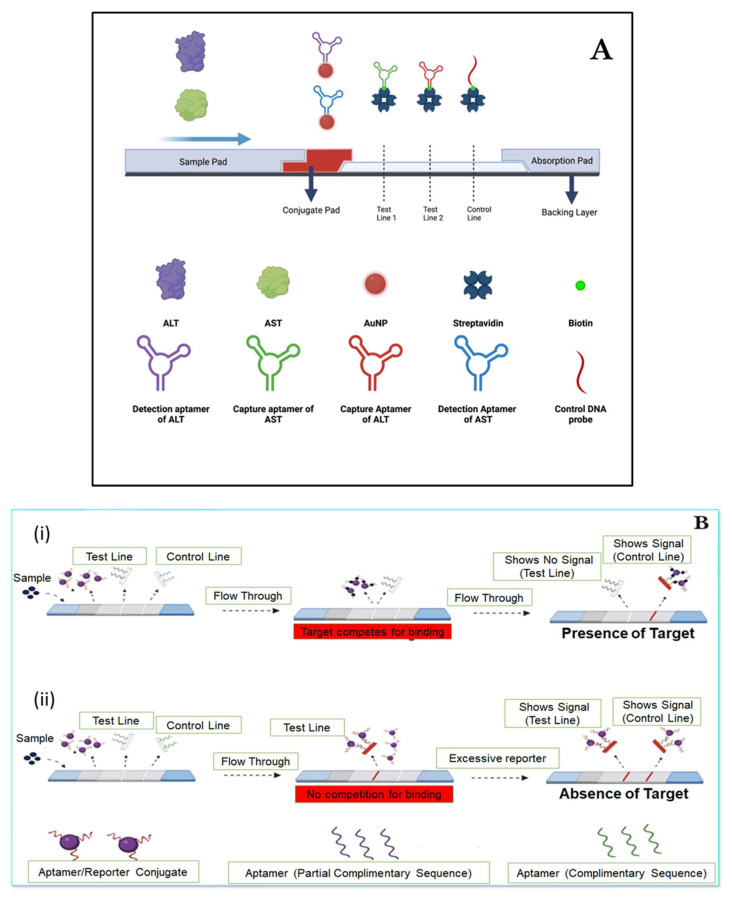
(**A**) Aptamer lateral flow assay using two different aptamers binding to the same targets (binding ratio of target to aptamer; 1:2). The test lines are prepared streptavidin-biotinylated capture aptamers. The capture aptamers are labeled with AuNPS dispensed on the conjugate pad. The sample with ALT and AST form sandwich complexes with both capture and detection aptamers, resulting in a signal in both test lines. The control line in general is a complete complement of detection aptamer sequences to validate the assay system. (**B**) Aptamer lateral flow assay using partial and complete complementary sequences with the binding ratio of target to aptamer is one. (i) In the presence of ALT/AST, the AuNP-labeled aptamer forms strong complexes with targets that will not form duplexes with partially complementary sequences, resulting in no/low signal in the test line. (ii) In samples without ALT/AST, the aptamers are free to form a duplex with both partial complementary and complete complementary sequences resulting in signals in both test and control lines. The control lines are labeled with a complete complement of aptamer sequences to validate the assay system.

High-affinity aptamers are initially used to develop a simple aptasensor for initial testing, with its performance determined by both the target recognition receptor and the transducer. The sensor’s signal response will be tested against variable concentrations of ALT and AST in the dynamic range of sub-pM to µM. In linear calibration plots, the concentration of target enzymes against the signal released is then obtained. The limit of detection (LOD) of each biosensor will be calculated from the given formula 3σ/b, where σ is the standard deviation of the blank measurements, and b is the slope of the straight line. The cross-reactivity of the aptamers will then be tested against other enzymes with similar structures and functions, confirming the specificity of the aptamer to the target enzymes even in the presence of other molecules. If the LOD of the sensor is satisfactory, the aptamers may be used to develop miniaturized POC testing instruments such as lateral flow assay without the need for any readout instruments [22]. The aptamers are relatively small compared to these enzymes; therefore, there is a possibility of two different aptamer binding sites existing for larger molecules, such as ALT and AST. Assuming two binding sites (enzyme-to-aptamer ratio: 1:2), we propose the design of an aptamer-based lateral flow sandwich assay, as shown in Figure 1A. On the other hand, if the enzyme has only one binding site for the aptamer (enzyme-to-aptamer ratio: 1:1), a sandwich assay cannot be used, so we suggest aptamer-based LFAs based on the one-site-binding mechanism, as depicted in Figure 1B.

A typical LFA consists of three major components, such as recognition, reaction, and signal transduction elements assembled on a paper-based strip. Five different parts are integrated with this strip, including a sample pad, conjugate pad, nitrocellulose membrane, absorbent pad, and backing plate. In general, the backing plate is the supporting layer, above which all the other components are assembled (overlapping each other) to ensure that the testing sample migrates over the strip. The sample pad is made up of a cellulose membrane, and the conjugate pad will be excessively loaded with the recognition element (the aptamer) labeled with a reporter (gold nanoparticles). When a liquid sample containing ALT and AST is loaded, the ALT/AST interacts with AuNP-labeled aptamers and moves to the test lines. The test lines are loaded with a second aptamer or a short complement of the first aptamer. Depending upon the nature of the aptamer target analytes’ complexation mechanism, the target analyte forms a sandwich complex, producing a signal in the test line. In the other design, however, the target analyte complex will not interact with the aptamer’s complementary sequence due to a less stable aptamer-cDNA duplex, and therefore, no signal will be generated [34]. The signal’s intensity is directly proportional to the amount of ALT/AST loaded. The liquid sample then moves further towards a control line containing a capturing agent that captures the excess aptamers, thus validating the performance of the LFA model.

In summary, we believe that aptamer-based lateral flow sensors would prove to be rapid, low-cost, and user-friendly, and can be used in the field without trained personnel. For immobilization, the dried reagents can be loaded on the chromatographic strip under optimized conditions and should be stored in the proper storage conditions for better results. Although the test is semiquantitative, the color density of the test line indicates the levels of ALT and AST and reflects the degree of liver damage. Many aptamer-based strip assays have been developed for the detection of several disease biomarkers. For example, Liu et al. developed a lateral flow thrombin aptasensor in which an AuNP-labeled detection aptamer and an immobilized capture aptamer in the test zone form a sandwich complex in the presence of thrombin. This strip-based assay can detect as low as 1.5 nM of thrombin within 10 min [34]. Quantitative detection of thrombin by aptamer-based LFA was achieved with a LOD of 0.85 nM within 10 min [51]. Therefore, we strongly believe that the selection of high-affinity, specific aptamers for ALT and AST, and using them to develop an aptamer-based point-of-care (APOC) device, would provide an affordable method to follow liver functions from home, which may prevent long-term liver damage.

## Figures and Tables

**Table 1:** Biosensors developed for the detection of ALT and AST.

Analyte	Detection Method	Material Used	Detection Range	LOD	Ref
ALT	Chronoamperometric	Membrane-coated immunosensors/self-assembled monolayer mediator-coated gold electrode	10 ng/mL–100 µg/mL	10 ng/mL	[36]
ALT	Dry-Chemistry Electrochemiluminescence	DC-ECL chip/detection layer (baseplate, electrode pad, and carrier pad)	5–50 U/L	1.702 U/L	[40]
ALT	Colorimetric	B-doped core–shell Fe@BC nanozyme	10–1000	4 U/L	[41]
AST	Electrochemical	Multilayered enzyme electrode by LBL technique	7.5–720 U/L in PBS 30–240 U/L in human plasma	13.9 U/L 20 U/L	[42]
AST	Electrochemical	Catalytic iridium nanoparticles dispersed on carbon paste	0–250 U/L	25.3 U/L	[43]
ALT	Electrochemical	Glutamate oxidase-modified platinum electrode	10–1000 U/L	3.29 U/L	[46]
ALT	Amperometric	Perm-selective polymer layers modified and glutamate oxidase immobilized micro-platinum wire microelectrode	10 to 900 U/L	8.48 U/L	[47]
ALT	LSPR	MoS2-NPs/CeO2-NPs	10–1000 U/L	10.61 U/L	[48]
ALT	Electrochemical	Microelectrode based on a molecularly imprinted pyruvate oxidase enzyme	25–700 U/L	2.97 U/L	[49]
AST/ALT	Hydrogel-Colorimetric	Au-decorated CoAl-layered double oxide (Au/LDO)	-	15 U/L (ALT) 10 U/L (AST)	[50]

## Data Availability

No additional supporting data available.
